# Reevaluation of Criteria and Establishment of Models for Total Thyroidectomy in Differentiated Thyroid Cancer

**DOI:** 10.3389/fonc.2021.691341

**Published:** 2021-09-09

**Authors:** Zhenghao Wu, Yunxiao Xiao, Jie Ming, Yiquan Xiong, Shuntao Wang, Shengnan Ruan, Tao Huang

**Affiliations:** Department of Breast and Thyroid Surgery, Union Hospital, Tongji Medical College, Huazhong University of Science and Technology, Wuhan, China

**Keywords:** thyroid cancer, thyroidectomy, preoperative, risk factors, model

## Abstract

**Introduction:**

After the publication of the 2015 American Thyroid Association (ATA) guidelines, the indication for total thyroidectomy (TT) was reported to be underestimated before surgery, which may lead to a substantial rate of secondary completion thyroidectomy (CTx).

**Methods and Materials:**

We retrospectively analyzed differentiated thyroid cancer patients from Wuhan Union Hospital (WHUH). Univariate analysis was performed to evaluate all preoperative and intraoperative factors. New models were picked out by comminating and arranging all significant factors and were compared with ATA and National Comprehensive Cancer Network (NCCN) guidelines in the multicenter prospective Differentiated Thyroid Cancer in China (DTCC) cohort.

**Results:**

A total of 5,331 patients from WHUH were included. Pre- and intraoperative criteria individually identified 906 (17.0%) and 213 (4.0%) patients eligible for TT. Among all factors, age <35 years old, clinical N1, and ultrasound reported local invasion had high positive predictive value to predict patients who should undergo TT. Accordingly, we established two new models that minorly revised ATA guidelines but performed much better. Model 1 replaced “nodule size >4 cm” with “age <35 years old” and achieved significant increase in the sensitivity (WHUH, 0.711 *vs.* 0.484; DTCC, 0.675 *vs.* 0.351). Model 2 simultaneously demands the presence of “nodule size >4 cm” and “age <35 years old,” which had a significant increase in the specificity (WHUH, 0.905 *vs.* 0.818; DTCC, 0.729 *vs.* 0.643).

**Conclusion:**

All high-risk factors had limited predictive ability. Our model added young age as a new criterion for total thyroidectomy to get a higher diagnostic value than the guidelines.

## Introduction

Differentiated thyroid cancer (DTC) is one of the most rapidly growing malignancies globally in recent years (1–3), and surgery plays a significant role in the treatment for DTC patients. Both the 2015 American Thyroid Association (ATA) and 2018 National Comprehensive Cancer Network (NCCN) revised the guidelines, which narrowed the indication of surgery for DTC and brought considerable controversies about the reasonable treatment for thyroid cancer (4, 5) (**Supplementary Table**). All those altering based no significant prognosis difference between total thyroidectomy (TT) and thyroid lobectomy (6, 7), the effectiveness of complete thyroidectomy (CTx, secondary surgery), and the cautious choice of iodine 131 treatment (8, 9). They recommended that TT is limited to fewer high-risk populations, including a family history of thyroid cancer, radiation history, extrathyroidal extension (ETE), tumor size >4 cm, and clinical lymph node metastasis (cN1). DTC patients without these high-risk factors first should undergo thyroid lobectomy. If postoperative pathology reported risk factors such as aggressive histology, these patients have to undergo secondary CTx. These guidelines presented surgeons a dilemma raised in previous studies that 30–40% of patients who were not eligible for TT need to undergo secondary surgery (10, 11). This brings potential patients’ complaints, economic losses, complications, and anesthesia risks.

Although the guidelines proposed many pre- and intraoperative factors, the accuracy and reliability of these factors to decide TT and eliminate the need for CTx have been studied less. Our study analyzed the clinical–pathological data of thyroid cancer patients from Wuhan Union Hospital (WHUH) and evaluated all the factors’ ability to predict reasonable TT. Finally, we tried to develop and validate new models to indicate TT by a new algorithm.

## Methods

### Clinical–Pathological Data 

The training set was from the Union Hospital of Tongji Medical College of Huazhong University of Science and Technology (Wuhan Union Hospital, WHUH) for retrospective analysis. WHUH database included registered thyroid cancer patients from 2008 to 2018. The validation cohort is from the Differentiated Thyroid Cancer in China (DTCC) study [registered at ClinicalTrials.gov (NCT02638077)], which included thyroid cancer patients from nine hospitals with high thyroid surgery volume from 2014 to 2016. All patients were confirmed DTC through pathology assessment of preoperative core needle puncture (CNP) or intraoperative frozen section, and thyroidectomy was completed by experienced thyroid surgeons. Our database recorded mainly, first, age at first diagnosis, gender, family history of malignant tumor, history of another malignant tumor; second, preoperative status of lymph nodes (LN) including central and lateral compartments. Clinical N1 refers to N1a (suspicious central LN metastasis) or (and) N1b (suspicious lateral neck LN metastasis). Third are the intraoperative evaluations recorded by the surgeons, including LN dissection scope and visual inspected tumor invasion. Fourth are the postoperative pathological data including tumor size, pathological subtypes, ETE (excluding capsule invasion), and number of involved LN.

**Figure 1:**
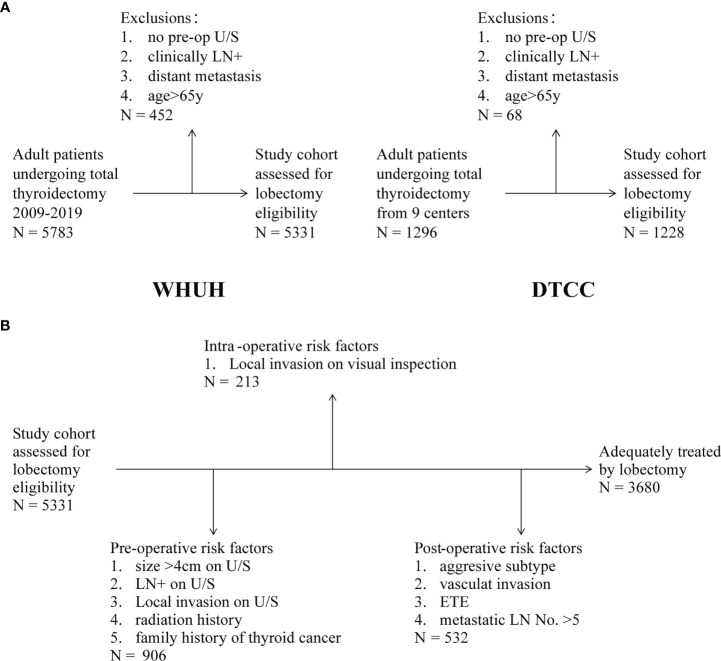
**(A)** Inclusion and exclusion criteria in WHUH database and DTCC cohort. **(B)** Patients with eligibilities to total thyroidectomy (TT) in the different evaluation stages.

The study inclusion criteria were adult patients (age ≥18 years and ≤65 years old at the date of surgery) who underwent TT and were confirmed DTC by the pathological diagnosis. We excluded any patient who did not have preoperative ultrasound reports and those with distant metastatic disease. Notability, maximum thyroid nodule size was not available in a few included patients’ ultrasound results. **Figure 1A** provides a flowchart showing the WHUH and DTCC screening.

The study was performed in accordance with the Declaration of Helsinki (as revised in 2013) and approved by the Ethical Committee of the Union Hospital, Tongji Medical College of Huazhong University of Science and Technology (No. 0304-01). The informed consent for data publication was not required for this study’s retrospective nature.

### Evaluation of Preoperative and Intraoperative Factors 

Univariate analysis was performed to identify the significant correlation between pre- or intraoperative clinical characteristics and high-risk pathological results. We applied two methods to evaluate the ability of each factor to predict the TT. First, the positive predictive value (PPV) indicates the likelihood that someone with preoperative high-risk factors actually should undergo the TT. Factors with high PPV can identify patients who require TT. Thus, it will be sufficient to follow up any positive result of these high PPV factors to obtain an accurate assessment of TT.

Second, all patients are divided into two groups according to whether they need TT based on postoperative ATA risk factors. We performed t-tests (continuous variable) or chi-square tests (categorical variable) for each pre- or intraoperative factor between these two groups. Significant factors (p < 0.001) were identified as effective criteria to distinguish whether patients require TT.

### Construction of New Predictive Models

After screening all factors through two kinds of univariate analysis above, several significant preoperative (Pre-op) or intraoperative (Intra-op) factors were selected for further model construction. Then, these significant factors were randomly arranged and combined through R programming to construct numerous multivariate models, containing one or all possible risk factors. The logical relationship between factors in models could be “AND” (true if both factors are true) and “OR” (true if either factor is true). Therefore, each model can be described as an expression, such as “① OR ②” and “① AND ②” (NCCN guidelines). In total, the R program randomly generated 3,840 models according to the above method. Then, we selected models in the top 10 percentile of both sensitivity and specificity among all models and sorted these models according to the area under curve (AUC). In summary, this study established the two models with the best performance. Internal sets from WHUH and external sets from DTCC were used to validate both new models, namely, ATA and NCCN guidelines.

### Statistical Analysis

The clinicopathological characteristics of patients in two databases were presented by t-test (continuous variable) or chi-square test (categorical variable). All statistical analyses were performed using SPSS version 23.0 (SPSS, Chicago, IL, USA) or R software version 3.2.1 (http://www.r-project.org). All p values were two-sided; p < 0.05 is considered statistically significant.

## Results

### Clinical–Pathological Data Including WHUH Database and DTCC Cohort

A total of 5,331 differentiated thyroid cancer patients were included after excluding 452 ineligible patients in the database from WHUH. As shown in **Table 1**, the average age was 43.8 ± 10.3 years old, and 4,126 (77.4%) were female. Meanwhile, there were 4,909 (92.1%) patients who underwent LN dissection, including 4,882 (91.6%) patients with central compartments and 1,019 (19.1%) patients with neck lateral compartments.

**Table 1:** Baseline characteristics in development cohort and validation cohort.

		Union Hospital	DTCC	*p*-value
**No. of patients**		5331	1228	
**Gender**	**Male**	1,198 (22.5%)	351 (28.6%)	0.001*
	**Female**	4,126 (77.4%)	877 (71.4%)	
	**Not known**	7 (0.1%)	0	
**Mean age, years**	**Years (SD)**	43.8 (10.3)	41.0 (10.5)	0.024*
**Family history of thyroid cancer**	**Yes**	46 (0.9%)	29 (2.4%)	0.001*
	**No**	5,285 (99.1%)	1,198 (97.6%)	
	**Not known**	0	1 (0.1%)	
**Clinical N stage**	**N1**	394 (7.4%)	470 (38.3%)	0.001*
	**N0**	4,937 (92.6%)	727 (59.2%)	
	**Not known**	0	31 (2.5%)	
**Maximum tumor size, cm**	**≤4**	5,041 (94.6%)	1,210 (98.5%)	0.583
	**>4**	36 (0.7%)	17 (1.4%)	
	**Not known**	254 (4.8%)	1 (0.1%)	
**LN metastasis, No.**	**0**	2,731 (51.2%)	239 (19.5%)	0.001*
	**1~5**	1,786 (33.5%)	562 (45.8%)	
	**>5**	814 (15.3%)	427 (34.8%)	
**Pathology ETE**	**Yes**	187 (3.5%)	336 (27.4%)	0.001*
	**No**	4,956 (93.0%)	892 (72.6%)	
	**Not known**	188 (3.5%)	0	

*p < 0.05.

SD, standard deviation.

In the DTCC cohort, 1,228 included patients underwent TT. The average age was 41.0 ± 10.5 years old. The percentage of female patients accounted for 71.4% (877), and 1,190 (96.9%) patients underwent LN dissection, among which 1,126 (91.7%) and 581 (47.3%) patients individually underwent central and neck lateral compartment LN dissection. Postoperative pathology revealed that ETE occurred in 336 patients (27.4%), and LN metastasis occurred in 989 patients (80.5%).

### Patients Eligible for Total Thyroidectomy

As shown in **Figure 1B**, the preoperative criteria identified 906 (17.0%) people eligible for TT, which consisted of 46 (5.1%) patients with family history of thyroid cancer, 484 (53.4%) patients with ultrasound (U/S)-reported tumor larger than 4 cm, 394 (43.5%) patients with clinical N1, and 57 (6.3%) patients with local invasion (including capsule invasion). A total of 3,014 (56.5%) patients need to undergo TT because of bilateral nodules according to the NCCN guideline. **Supplementary Figure 1** shows the composition relationship between preoperative risk factors. In the remaining 4,425 patients who were prepared for lobectomy, 213 (4.0%) patients were transferred to TT because of local invasion on visual inspection during operation. Results of the intraoperative frozen sections were not considered because of their overlap with postoperative pathology reports. In summary, in our WHUH database, preoperative clinical characteristics and intraoperative findings can identify 1,119 (21.0%) patients, and the remaining 4,212 patients were eligible for thyroid lobectomy. However, the postoperative pathological results showed that 12.6% (532/4,212) patients had indications of CTx theoretically for high-risk factors after thyroid lobectomy. For instance, 79 (1.9%) patients had ETE (muscle, recurrent laryngeal nerve, and blood vessel), and 1,914 (45.4%) “lobectomy” patients had LN metastasis, of which 467 (11.1%) patients had metastatic LNs >5. Finally, after identifying the pathology, it is sufficient for 31.0% (1,651/5,331) of the patients to perform TT.

In the overall cohort from WHUH, the rate of LN metastasis was 48.8% (2,600/5,331), the proportion of >5 metastatic LNs was 15.3% (814/5,331), the proportion of ETE was 3.5% (187/5331), and 947 (17.8%) patients were identified with intermediate-/high-risk thyroid cancer according to postoperative pathological diagnosis. Therefore, 62.9% (704/1,119) of patients who underwent TT were only identified as low-risk thyroid cancers after the surgery according to the ATA guideline.

### Evaluation of Pre-/Intraoperative Factors Based on Postoperative Pathology 

We evaluated the prediction ability of several pre-/intra-operative factors for each postoperative risk factor in the ATA or NCCN guidelines. First, among 1,108 patients with age <35 years old, there were 799 patients with LN metastasis, of which 370 patients had >5 metastatic LNs. Tumor size >4 cm and ETE were individually found in 12 and 34 patients. In total, 388 patients belonged to the high-risk subtype based on ATA guidelines. Second, in 394 patients with clinical N1, 341 patients were confirmed LN metastasis in the postoperative pathology, of which 228 patients had > 5 metastatic LNs. Other eight patients belonged to the high-risk subtype because of other factors. Third, 57 patients reported that thyroid tumors invaded the capsule or extraglandular tissues by U/S. Only 10 patients had ETE among 29 patients with the ATA risk factors in the postoperative pathology. In comparison, 45 patients had LN metastasis, of which 21 patients had metastatic LNs >5. Fourth, among 46 patients with a family history of thyroid cancer, 12 patients were classified into the intermediate-/high-risk group, including 9 patients with metastatic LNs >5, 3 patients with ETE, and 1 patient with tumor >4 cm (5). In 484 patients with thyroid nodules >4 cm reported by U/S, only 20 patients had tumor nodules >4 cm in postoperative pathology, 33 patients had ETE, and 90 patients had metastatic LNs >5. In total, 112 patients were identified with intermediate-/high-risk thyroid cancer according to ATA.

**Figure 2:**
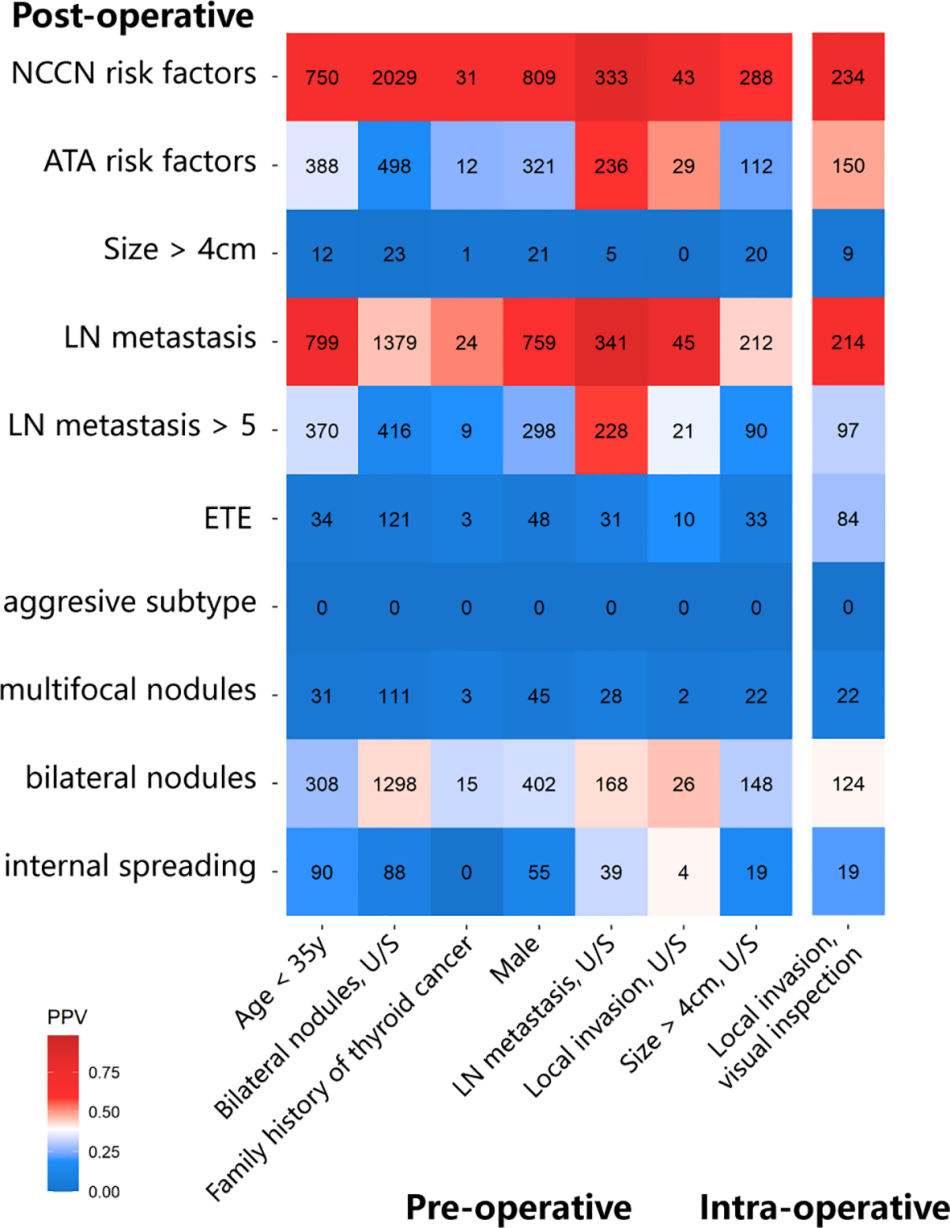
The heatmap shows the positive predictive value (PPV) of pre-/intra-operative factors to predict each postoperative intermediate-/high-risk factor.

Positive predictive value (PPV) was used to evaluate pre- and intraoperative characteristics and is shown in the heat map (**Figure 2**). Several factors were found to be a good predictor of TT based on the ATA guideline, including age <35 years (PPV, 35.0%), clinical N1 (PPV, 59.9%), and U/S-reported local invasion (PPV, 50.9%). However, some guideline suggested factors that performed unsatisfactorily, such as family history of thyroid cancer (PPV, 26.1%), bilateral nodules (PPV, 16.3%), and nodule size >4cm (PPV, 22.4%).

### Construction of Risk Model and Validation in External Cohort

**Figure 3:**
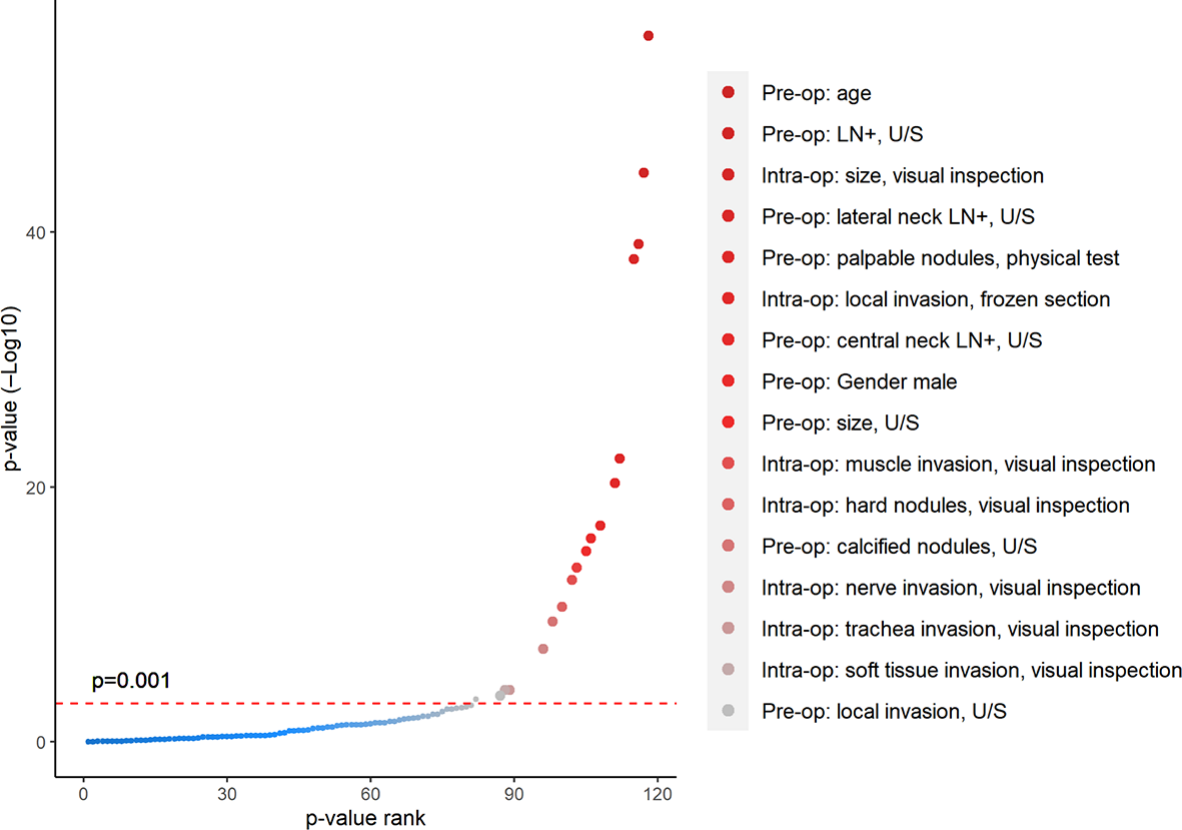
Univariate analysis showed significant correlations between clinical characteristics and high-risk pathological results. All patients are divided into two groups according to whether they need the TT based on postoperative ATA risk factors. p-value was calculated for each pre-/intraoperative factor through t-test (continuous variable) or chi-square test (categorical variable) between these two groups. All factors were ranked along the X-axis from larger to smaller p-values, with significant factors shown in red dots (p < 0.001).

Univariate analysis showed (**Figure 3**) that a large number of potential factors had significant associations with intermediate- or high-risk thyroid cancer, including demographic data (age and gender), preoperative ultrasound (tumor size, tumor calcification, local invasion, and suspicious central/lateral compartment LN metastasis), and intraoperative ETE (all *p* < 0.001). After selections through univariate analyses above, eight preoperative (Pre-op) or intraoperative (Intra-op) factors were identified with good predictive ability to TT, including (1) Pre-op, age < 35 years old; (2) Pre-op, bilateral nodules, U/S; (3) Pre-op, male gender; (4) Pre-op, LN metastasis; (5) Pre-op, size > 4 cm, U/S; (6) Pre-op, family history of thyroid cancer; (7) Pre-op, local invasion, U/S; and (8) Intra-op: local invasion and visual inspection.

**Figure 4:**
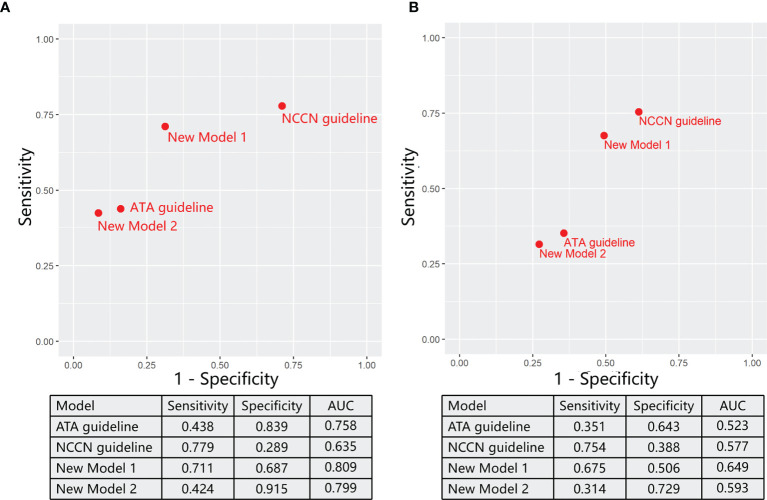
The ability of varying models for predicting reasonable total thyroidectomy in the training sets **(A)** and validation sets **(B)**.

Then, we evaluated two models from ATA and NCCN guidelines to predict the TT before the end of surgery. ATA models were defined as “(4) OR (5) OR (6) OR (7) OR (8)” through logical expression. In the training sets (**Figure 4A**), the ATA model performed well in specificity (0.839) but unsatisfactory in sensitivity (0.438). Compared with the ATA model, the NCCN model supplements factor (3) as the indication of TT, which also expressed as “(3) OR (4) OR (5) OR (6) OR (7) OR (8)”. Then, specificity (0.289) drops sharply in spite of a relative increase in sensitivity (0.779).

In order to obtain models with better performance than guidelines, we randomly arrange and combine eight significant factors as reported in the method section and thus picked out two models through comprehensively evaluating sensitivity, specificity, and AUC (**Figure 4A**). On the basis of ATA guidelines, Model 1 (① OR ④ OR ⑤ OR ⑥ OR ⑦ OR ⑧) replaced low PPV factor (⑤) with high PPV factor (①) as the standard of TT. Model 1 achieved a significant increase in the sensitivity (0.711) and a minor decrease in the specificity (0.687) compared with ATA guidelines. In Model 2 [(④ OR (① AND ⑤) OR ⑥ OR ⑦ OR ⑧], when other factors in the ATA guideline did not exist, patients underwent the TT only if both two factors (① AND ⑤) are true. Model 2 had a significant increase in the specificity (0.915) and a minor decrease in the sensitivity (0.424) compared with ATA guidelines.

Finally, we assess these models in an external validation cohort from the DTCC project (**Figure 4B**). The sensitivity of ATA and NCCN guidelines is individually 0.351 and 0.754, and the specificity is 0.643 and 0.388, respectively. Consistent with the training sets, new model 1 performed well in sensitivity (0.675), and model 2 was good at specificity (0.729). Notability, both new models 1 (0.649) and 2 (0.593) achieved increased AUC than ATA (0.523) and NCCN (0.577) guidelines.

## Discussion

The surgical scope to thyroid cancer had experienced a process from “large to small” (10–12). Concerns about overtreatment further limited total thyroidectomy (TT) and prophylactic central LN dissection in DTC patients. The guidelines changed indications for TT based on evidence from the National Cancer Data Base (NCBD) and the Surveillance, Epidemiology, and End Results (SEER) database of the United States (6, 7, 13). However, the predictive ability of these preoperative risk factors for reasonable TT needs to be reexamined, and studies for evaluating preoperative TT indication based on large and multicenter cohorts were rare. Our retrospective study, which included the most extensive samples in China, evaluated the ability of pre-/intraoperative factors as indications for TT and developed new diagnostic models that were validated well in the DTCC cohort. In our database from WHUH, 12.6% of patients with low-risk DTCs initially meeting the criteria for lobectomy would ultimately require their entire thyroid to be removed. In contrast, 62.9% of patients who received TT because of pre-/intraoperative risk factors were found to be overtreated after the surgery.

### Preoperative Lymph Node Metastasis

In all preoperative risk factors, clinical N1 had the highest PPV for predicting the intermediate–high-risk thyroid cancer. In our study, in 394 patients with U/S reported cN1, 86.5% of patients were confirmed to have LN metastasis, and about 70% of the patients were intermediate-/high-risk patients. Meanwhile, cN1 can predict metastatic LN >5 (57.87%), which was a leading cause for CTx after lobectomy. LN metastasis is an independent risk factor of the prognosis of thyroid cancer patients (14, 15). As an indispensable role in predicting TT, the status of LN was most likely to be underestimated in patients thought to be eligible for lobectomy. U/S is a widely used method to diagnose cN1 (16), but it is challenging to detect metastatic LNs in the central compartment. As a supplement, previous studies had developed several models for predicting LN metastasis, which indicated body mass index (BMI), age, and tumor size as potential risk factors (17, 18). However, the actual predictive value needs more validations. In addition, intraoperative lymph node frozen inspection was not applied in the majority of Chinese hospitals, and previous research recommended that it should be regarded as clinical LN metastasis and be applied as effective criteria for intraoperative conversion from lobectomy to TT (11). In our cohort, a high proportion of metastatic LNs was detected due to a high rate of prophylactic central LN dissection in the past 10 years. However, it is worth to be noted that prophylactic central LN dissection may significantly increase the rate of temporary recurrent nerve injury and hypoparathyroidism, especially in the older group (19). Intraoperative neuro-monitoring, with good sensitivity and negative predictive value, may detect proximal recurrent nerve injury. Oral calcium and vitamin D supplements were able to prevent laboratory hypocalcemia and hypocalcemia symptoms for transient parathyroid gland injury (20, 21).

### Tumor Size

The tumor size constitutes an essential part of the indication for TT, while T stage was also an independent risk factor for both survival and recurrence (22, 23). In our results, tumor size may be the most overestimated preoperative risk factor. Patients with nodule size >4 cm accounted for 53.4% of all patients who were eligible for TT, the highest percentage among all preoperative factors. However, the postoperative pathology showed that only 4.1% of patients had tumor size more than 4 cm, and only 23.1% of patients were classified into intermediate–high risk. The main reason is that U/S often reports a larger tumor size compared with pathology sections (24, 25). Deveci et al. also reported that the concordance of thyroid nodule >1.0 cm measured by U/S and gross pathology examination is ≤50% (25).

### Other Factors

Although U/S has a high sensitivity for the detection of ETE (26), it is difficult to distinguish between ETE and capsular invasion. Therefore, preoperative capsular invasion under U/S was also considered to be eligible for TT. Thyroid cancers with only capsular or the perithyroidal soft tissue invasion were classified as low risk because of their minimal prognosis influence (27, 28). In our study, 45 patients were found to have LN metastasis among 57 patients with U/S-reported invasion. Our finding also corroborated the previous results that ETE were associated with LN metastasis (29). In addition, thyroid cancer family history had an unsatisfactory PPV for intermediate–high-risk thyroid cancer. Relatives with low grades of thyroid cancer (ep. microcarcinoma) or distant relationship might rarely influence the patient (30).

### A New Algorithm for Constructing Models 

In our database, we also presented the differences of ATA and NCCN guidelines. In both WHUH database and DTCC cohort, ATA guidelines had higher specificity compared with NCCN guidelines, which means that patients eligible for lobectomy are more likely to have low-risk characteristics. However, NCCN has a higher sensitivity, owing to contralateral thyroid nodules as one of the TT criteria. It has been reported that 16%–30% of the patients with bilateral nodules were diagnosed with incident malignant contralateral tumor (31–33). According to the ATA guideline, 17.16% of patients need TT at the first surgery in our database. However, the proportion of patients who need TT would increase by more than three times (56.89%) if NCCN criteria were rigorously implemented.

Our study developed a new algorithm for constructing models. We randomly arranged and combined all significant clinical factors into numerous models. Then, models with good performance were selected, compared with old guidelines in the training set, and validated in DTCC cohorts. The major pros and cons of this new algorithm are as follows: (1) new models are expressed as logical relationships (“AND” and “OR”), which have similar structures with guidelines and are suitable for clinical application. In contrast, scoring systems such as nomograms require much mathematical calculation. (2) All possible combinations of these risk factors were automatically generated and filtered through computer programs. No models will be missed like forward or backward methods. (3) This algorithm can only be applied for categorical variables, while threshold values should be set for continuous variables. Notably, almost all risk factors for predicting TT were categorical variables.

The main change in the new model was age, as the threshold value 35 years old was from the largest AUC, which leads the new model to obtain higher sensitivity and specificity. Young age was found highly related to intermediate–high thyroid cancer in previous retrospective studies (34, 35), which suggested a high risk of recurrence. Hye-Seon Oh et al. also found that young and male patients should be recommended active surgery for more frequent large-volume LNM (36). Age is an essential factor influencing the prognosis of thyroid cancer. Thyroid tumors in the younger patients (<25 years old) and the older (>65–70 years old) group had been reported to have a more invasive behavior, which seems rational to undergo central LN dissection. However, it deserved personalized processing to balance the risk and quality of elderly patients’ life after prophylactic central LN dissection (19). Meanwhile, all the evidence between the clinical model and invasive differentiated thyroid cancer need validation from molecular diagnosis and mechanism experiments (37).

### Limitation

First, the proportion of preoperative intermediate–high-risk patients may be underestimated because potential risk factors like neck radiation history were not fully recorded in the WHUH database. Although we have recorded radiotherapy history, it is difficult for retrospective studies to obtain the history of neck radiation examination. Second, some aggressive histology subtypes (ep. hobnail variant of PTC) were not reported by postoperative pathology, leading to underestimating the proportion of high-risk patients. Third, the lack of molecular markers of thyroid cancer hinders the preoperative decision on the resection scope of thyroid cancer. More preoperative serum results such as platelet counts and thyroid autoantibodies are correlated with recurrence of thyroid cancer, which would be potential parameters for managing total thyroidectomy in the future (38, 39).

## Conclusion

Age <35 years old, LN metastasis, and U/S reported local invasion was found to be a good predictor of total thyroidectomy (TT) based on the ATA guideline. Our model added young age as a new criterion for TT and had a higher diagnostic value in the training and validation cohort.

## Data Availability Statement

The raw data supporting the conclusions of this article will be available under reasonable requests.

## Ethics Statement

The studies involving human participants were reviewed and approved by Ethical Committee of the Union Hospital, Tongji Medical College of Huazhong University of Science and Technology (No. 0304-01). Written informed consent for participation was not required for this study in accordance with the national legislation and the institutional requirements.

## Author Contributions

ZW and YXX conceived of the study and analysis plan. JM, YQX, SW, and SR collected data. ZW analyzed the data. YXX wrote the first draft of the manuscript. TH had full access to all the data in the study and had final responsibility for the decision to submit for publication. All authors contributed to the article and approved the submitted version.

## Conflict of Interest

The authors declare that the research was conducted in the absence of any commercial or financial relationships that could be construed as a potential conflict of interest.

## Publisher’s Note

All claims expressed in this article are solely those of the authors and do not necessarily represent those of their affiliated organizations, or those of the publisher, the editors and the reviewers. Any product that may be evaluated in this article, or claim that may be made by its manufacturer, is not guaranteed or endorsed by the publisher.

## Supplementary Material

Supplementary Figure 1 The number of patients with one or more preoperative factors.
