# Supplementary figures and images for: Reevaluation of Criteria and Establishment of Models for Total Thyroidectomy in Differentiated Thyroid Cancer
Source: Front Oncol. 2021 Sep 9;11:691341. doi: 10.3389/fonc.2021.691341 (PMC8458835; doi:10.3389/fonc.2021.691341)

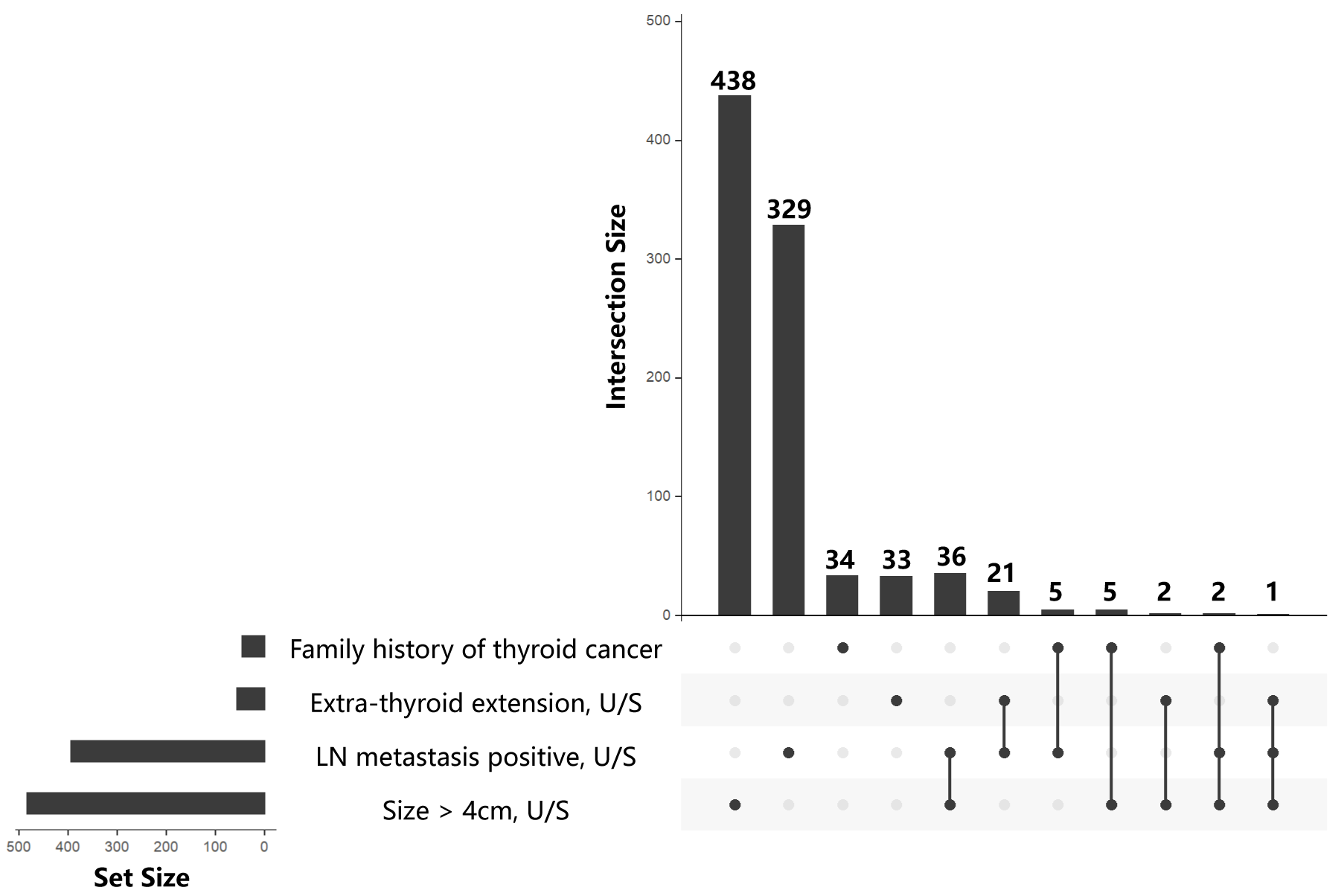

Supplement: Supplementary Figure 1 — The number of patients with one or more preoperative factors. [file Image_1.tif]
