# Supplementary material for: Reevaluation of Criteria and Establishment of Models for Total Thyroidectomy in Differentiated Thyroid Cancer
Source: Front Oncol. 2021 Sep 9;11:691341. doi: 10.3389/fonc.2021.691341 (PMC8458835; doi:10.3389/fonc.2021.691341)
Supplement: Supplementary file 2 [file Table_1.docx]

| **Supplementary Table: Total thyroidectomy criteria and Intermediated or high-risk factors from two guidelines.** | | | | | | |
| --- | --- | --- | --- | --- | --- | --- |
|  |  | **ATA** **criteria for TT** | **NCCN criteria for TT** | **NCCN criteria for CTx** | **Intermediated or high-risk factors (ATA)** | **Occult intermediated-high-risk thyroid cancer** |
| **Preoperative clinical characteristics** | **Family history of thyroid cancer** | **Yes** | **Yes** |  |  | **No** |
|  | **History of neck radiation** | **Yes** | **Yes** |  |  | **No** |
|  | **Thyroid tumor dimeter > 4cm** | **Yes** | **Yes** |  |  | **No** |
|  | **Cervical lymph node metastases (cN1)** | **Yes** | **Yes** |  |  | **No** |
|  | **Bilateral thyroid nodules (from NCCN)** | **None** | **Yes** |  |  | **No** |
| **Postoperative pathological**  **results** | **Tumor >4 cm** |  |  | **Yes** | **Yes** | **Yes or (and)** |
|  | **Positive resection margins** |  |  | **Yes** | **Yes** | **Yes or (and)** |
|  | **Gross ETE** |  |  | **Yes** | **Yes** | **Yes or (and)** |
|  | **Macroscopic multifocal disease (> 1 cm)** |  |  | **Yes** | **None** | **Yes or (and)** |
|  | **Lymph nodal metastasis*** |  |  | **Yes** | **Yes** | **Yes or (and)** |
|  | **Vascular invasion** |  |  | **Yes** | **Yes** | **Yes or (and)** |
|  | **Aggressive Histology** |  |  | **No** | **Yes** | **Yes or (and)** |
| **cN1: Clinical lymph node metastasis; TT: Total Thyroidectomy; CTx: Complete Thyroidectomy** | | | | | | |
| **ATA: American Thyroid Association NCCN: National Cancer Comprehensive Network** | | | | | | |
